# Supplementary material for: Epidemiology of Pneumococcal Pneumonia in Louisville, Kentucky, and Its Estimated Burden of Disease in the United States
Source: Microorganisms. 2023 Nov 20;11(11):2813. doi: 10.3390/microorganisms11112813 (PMC10673027; doi:10.3390/microorganisms11112813)

Supplementary Material

Title: Epidemiology of Pneumococcal Pneumonia in Louisville, Kentucky and Its Estimated Burden of Disease in the United States

Section Number	Section Title	Page Number
1	Participating Hospitals	1
2	Screening Criteria	4
3	Incidence Calculations	5
4	Geospatial Methods	9
5	Mortality Extrapolated to the U.S. Population Hospitalized with CAP	11
6	Data Quality	12
7	Additional Supplemental Tables	13

1. Participating facilities

A total of nine hospitals serve the adult population of Louisville, Kentucky. All nine facilities were included in the University of Louisville Pneumonia Study. Information regarding each participating institution is as follows:

A. University of Louisville Hospital

The University of Louisville Hospital has evolved from a county hospital that started in 1823 with 150 beds to a state-of-the-art complex offering a complete range of inpatient and outpatient services. It is the primary adult teaching hospital for the University of Louisville's Health Sciences Center. With 404 licensed beds, the tertiary acute-care hospital provides a full range of diagnostic, therapeutic, emergency, and surgical services, including the region's only Level 1 adult trauma center.

B. Robley Rex VA Medical Center

The Robley Rex Veterans Administration (VA) Medical Center is a 120-bed acute care hospital located in Louisville, Kentucky. The VA hospital is one of the primary teaching hospitals of the University of Louisville Medical School.

C. Baptist Healthcare Hospital

With Joint Commission accreditation, this facility has specialized services for women's health, cancer, heart, orthopedics, neurosciences, emergency care, rehabilitation, sleep disorders, occupational health, and behavioral health, including psychiatric and chemical dependency care. This facility is licensed for 407 beds.

D. Norton Audubon Hospital

Norton Audubon Hospital offers a full range of highly specialized medical and surgical services, including an internationally recognized heart institute and newly expanded emergency

department. This facility is licensed for 432 beds. Pneumonia is within their top 10 most common diagnoses.

E. Norton Suburban Hospital

Norton Suburban Hospital offers more than 1,200 physicians to patients in and around Louisville. Norton Suburban Hospital offers a full range of diagnostic, therapeutic, emergency and surgical services and is licensed for 343 beds.

F. Norton Downtown Hospital

Norton Hospital Downtown is focused on specialty surgical services, advanced diagnostics, oncology, orthopedics and maternal-fetal medicine. The hospital has served as a teaching facility for the University of Louisville School of Medicine for over 50 years. This facility is licensed for 955 beds and has all private rooms, higher cancer survival rates than national average, and advanced diagnostics and surgical technology.

G. Jewish Hospital

Jewish Hospital is a 442- bed tertiary medical center, which encompasses virtually every aspect of healthcare. Jewish Hospital is one of the premier heart hospitals in the U.S., dedicated to excellence in clinical care, research and education, and is a leader in Kentucky, being one of the first to achieve Chest Pain Accreditation. Jewish Hospital has been the site of many "firsts," including the world's first and second successful AbioCor® artificial heart implants and the world's first hand transplant. Jewish Hospital is also federally designated to perform all five solid organ transplants–heart, lung, liver, kidney and pancreas.

H. Saints Mary and Elizabeth Hospital

Saints Mary and Elizabeth Hospital is a 298-bed facility. It is Louisville's premier Catholic care giving facility. This facility offers many services, including surgical services, a state-of-the-art

wound healing center, diagnostic imaging, cancer care, outpatient chemotherapy, cardiac services and a 20-bed emergency room.

I. Norton Brownsboro Hospital

Norton Brownsboro Hospital is Louisville's newest hospital and offers a full range of inpatient and outpatient cancer services; cardiovascular and pulmonary services; neuroscience services; orthopedics and spine services; women's services; and additional services such as emergency medicine, endoscopy, outpatient diagnostic services, physical/speech/occupational therapy and surgical services.

The University of Louisville Pneumonia Study was approved by each hospital prior to enrolling patients.

2. Screening criteria

All adult patients hospitalized were pre-screened for participation in the study using the following criteria: a chief complaint of cough, shortness of breath, shortness of air, fever, chills, malaise, or chest pain, or an admitting diagnosis of community-acquired pneumonia, pneumonia, altered mental status, confusion, fall, seizures, aspiration, cerebrovascular disease, transient ischemic attack, leukocytosis, leucopenia, respiratory failure, congestive heart failure, or urinary tract infection. Patients with at least one positive pre-screening criterion were screened. The screening criteria included a presence of a pulmonary infiltrate at chest x-ray or computed tomography scan of the chest. Patients with a pulmonary infiltrate were evaluated for inclusion into the study.

Inclusion criteria for the study were as follows: (1) presence of a new pulmonary infiltrate on chest radiograph and/or chest computed tomography scan at the time of hospitalization, defined by a board-certified radiologist's reading; (2) at least 1 of the following: (a) new cough or increased cough or sputum production, (b) fever >37.8°C (100.0°F) or hypothermia <35.6°C (96.0°F), (c) changes in leukocyte count (leukocytosis: >11000 cells/μL; left shift: >10% band forms/mL; or leukopenia: <4000 cells/μL); and (3) no alternative diagnosis at the time of hospital discharge that justified the presence of criteria 1 and 2.

3. Incidence calculations

a. *Calculating the 2014 Age-Specific Population in Louisville from U.S. Census Data*

For rate denominator data (unless otherwise specified), the 2014 American Community Survey was used. Since the 2014 denominator data for Louisville was not directly available via the American Community Survey, it was extrapolated as follows: Using 2010 age-specific U.S. Census Data for Louisville from the 2010 Decennial Census, we calculated the proportion of the population for each year of age (beginning with those 18 years of age) from table PEP_2014_PEPSYASEXN[1]. Next, we multiplied the proportion obtained in the previous step by the total 2014 adult Louisville population obtained from the U.S. Census Bureau projections (n=587,499). This provided the 2014 estimated adult population per each year of age for Louisville. The limitation of this approach is the assumption that the age-specific populations were proportionally equivalent in 2010 and 2014.

b. *Number of Patients Hospitalized with Pneumococcal Pneumonia*

As each patient hospitalized has a chance of being hospitalized with *S. pneumoniae* as an etiologic agent, it is intuitive that the number of patients hospitalized with pneumococcal pneumonia follows a Binomial distribution. Our study features two distinct patient groups, one with UAD-24 testing performed and one without. As each hospitalization is an independent event and the probability of being hospitalized due to pneumococcal pneumonia should not differ depending on whether or not a patient had UAD-24 testing performed, we can construct the following:

$$X_1 \sim Bin(n_1, p)$$

[1] Annual Estimates of the Resident Population by Single Year of Age and Sex for the United States: April 1, 2010 to July 1, 2014. Source: U.S. Census Bureau, Population Division. Release Date: June 2015. Accessed Feb 16, 2023. https://www2.census.gov/programs-surveys/popest/tables/2010-2014/national/asrh/PEPSYASEXN.pdf

$$X_2 \sim Bin(n_2, p)$$

$$Y = X_1 + X_2 \sim Bin(n_1 + n_2, p)$$

Where X_1 represents the number of patients hospitalized with pneumococcal pneumonia in the cohort of patients with UAD-24 testing (n_1), X_2 represents the number of patients hospitalized with pneumococcal pneumonia in the cohort of patients without UAD-24 testing (n_2), and Y represents the number of patients hospitalized with pneumococcal pneumonia in the whole study cohort, and p represents the probability of a CAP hospitalization being due to *S. pneumoniae.* Of these, only X_1, n_1, and n_2 are observed by our study. We estimated p as $\hat{p} = X_1/n_1$, with 95% confidence interval calculated by the Wilson score interval[2]. We estimated X_2 by the quantity $n_2\hat{p}$, and Y by the quantity $(n_1 + n_2)\hat{p}$, calculating confidence intervals using the upper and lower bound of $\hat{p}$.

c. *Annual Cumulative Incidence of pneumococcal pneumonia per 100,000 adults*

As Y calculated in section *b* is the total over two study years, we divided Y by two to get the annual number of patients hospitalized with pneumococcal pneumonia. Using this number, we divided by the population 2014 population of Louisville described in section *a* (n=587,499) and multiplied by 100,000 to get the rate per 100,000 adults.

d. *Comorbid Condition and Smoking Status-Specific Cumulative Incidence Rate Calculations*

The annual cumulative incidence of patients hospitalized with CAP in the city of Louisville by comorbid conditions and smoking status was calculated first by estimating the number of patients with pneumococcal pneumonia for each comorbid condition. We used the same

[2] L.D. Brown, T.T. Cai and A. DasGupta, Interval estimation for a binomial proportion (with discussion), *Statistical Science*, 16:101-133, 2001.

methodology as described in section *b*, except X_1, n_1, and n_2 were calculated by stratifying the whole study population to just those patients with a specific comorbid disease to produce X_{i1}, n_{i1}, and n_{i2} to then determine $\hat{p}_i$ and Y_i. To calculate the denominator for such patients, comorbid condition and smoking-specific denominators for these calculations were derived by multiplying the estimated prevalence of each condition/smoking status by the 2014 estimated adult Louisville population (methods outlined in section *a,* above). The prevalence multipliers were obtained from the Louisville 2014 Behavioral Risk Factor Surveillance System (BRFSS) data granted by the Louisville Metro Department of Public Health and Wellness. The only exception to this approach was for COPD. A modified formula was used to obtain the estimated adult population with COPD in Louisville since BRFSS surveys adults for the presence of chronic bronchitis, emphysema, and COPD combined. To include a similar denominator to our numerator (e.g. adult cases with COPD or emphysema, not necessarily only chronic bronchitis), we derived the COPD prevalence multiplier using data from BRFSS as well as from the 2014 National Health Interview Survey (NHIS). First, a Louisville/U.S. BRFSS adult Lung Disease ratio was computed. The BRFSS survey item related to COPD is the same in the U.S. as for Louisville and includes data for adults with chronic bronchitis, emphysema, and COPD combined. For Louisville, the adult lung disease prevalence was 9.16%, while for the U.S. it was 6.5%. Dividing these resulted in a value of 1.4092, suggesting that Louisville has approximately a 41% higher prevalence of adults with lung disease than the U.S. Second, we calculated the adult COPD/Emphysema (excluding chronic bronchitis only) prevalence in the U.S. from 2014 NHIS data. This value was 3.5984%. Finally, we increased this value by 40.92% to derive the estimated prevalence of adult COPD and Emphysema in Louisville, 5.0709%. All prevalence

values and estimated populations used for the denominator of the rate calculations can be found in Table S1 below.

Table S1: Multipliers for calculating comorbid-specific populations and 2014 estimated populations by comorbid conditions in Louisville, Kentucky

Variable	Behavioral Risk Factor Surveillance System Prevalence, 2014	Estimated Louisville Population, 2014**
COPD/Emphysema	0.050709*	29,792
Congestive Heart Failure	0.0523	30,727
Stroke	0.04	23,500
Diabetes	0.1145	67,269
Current Smoker	0.2427	142,587
Obesity	0.33	193,875

* Value computed using alternate formula described in the text

** Estimated denominator derived by multiplying the estimated prevalence by the estimated 2014 Louisville population of 587,499 adults ≥18 years of age.

For each comorbid condition, the corresponding Y_i was divided by two to get the yearly estimated number of patients with pneumococcal pneumonia, and then divided by the derived population with comorbid condition, and multiplied by 100,000 to produce the comorbid condition specific incidence per 100,000 adults (with comorbidity).

e. *Extrapolations to the United States*

Using the same table from section *a*, the total number of adults in the United States was calculated (n= 245,273,438). Using the assumption that the incidence rate in Louisville would be the same as the incidence rate in the United States, we calculated the annual number of adults hospitalized with pneumococcal pneumonia using the annual number of estimated adults with pneumococcal pneumonia ($^{Y}/_{2}$) divided by the number of adults in Louisville (n=587,499), times the number of adults in the United States.

4. Geospatial methods

The home address of each patient enrolled in the study was collected. An approximate latitude and longitude of the residence was obtained through the U.S. Census Bureau website which geomasked addresses through their geocoder tool. We used the census tract level for geospatial methods. Census tracts are subdivision of the U.S. Census Bureau data that account for a similar number of individuals with relative homogeneous socioeconomic indicators. Geospatial analyses were conducted using ArcGIS v10.2.

A kernel density heat map was created using location of each unique patient's home address at the time of first hospitalization each study year. Heat maps of Louisville were developed using census tract-level data representing the distribution of income, Black/African American, and elderly populations by census tract. To create heat maps this way, census tract centroids were used, with a population field equal to the number of individuals of the category, and a computed density area of 2 miles. Areas of high risk of hospitalization due to CAP were calculated using Kulldorff's Spatial Scan Statistic[3]. Using SaTScan software, we used a purely spatial Poisson model without covariates. Census tract data were imported into SaTScan, with cases defined as census tract aggregates of adults hospitalized with *Streptococcus pneumoniae* CAP, aggregates of the adult population per census tract, with longitude and latitude of census tract centroids to determine location.

Using the latitude and longitude, we were able to aggregate the number of adults hospitalized with *Streptococcus pneumoniae* per census tract and report census-tract specific rates using the number of patients included in the study as the denominator. A Local indicators of spatial

[3] Kulldorff M. A spatial scan statistic. *Communications in Statistics - Theory and Methods.* 1997;26(6):1481-1496.

association (LISA) map, using inverse distance weights, was produced to identify clusters of adults hospitalized due to *Streptococcus pneumoniae* CAP is depicted as **Figure S1**, below.

Figure S1. LISA map depicting proportion of adults hospitalized with *Streptococcus pneumoniae* CAP from adults hospitalized with CAP.

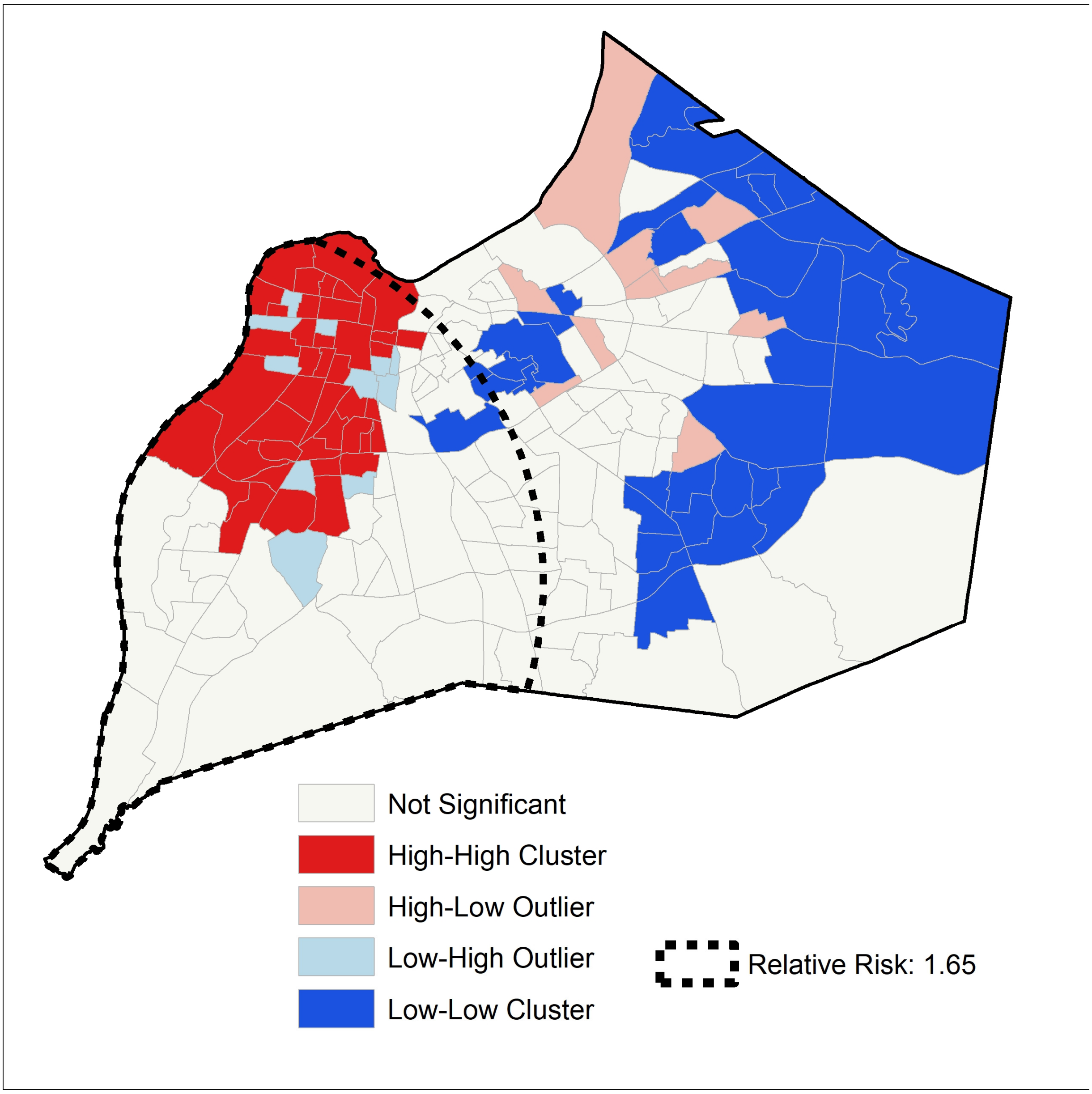

5. Mortality extrapolated to the U.S. population hospitalized with CAP

To estimate the number of individuals in the United States who will die annually during hospitalization, 30 days after admission, six months after admission, and one year after hospital admission for community-acquired pneumonia, the following methods were used. First, we estimated the total number of individuals in the United States expected to be hospitalized annually due to community-acquired pneumonia. To do this, we used the number of adult hospitalized patients in the United States from section *4.e* and multiplied this by observed proportion of unique patients who died in our study.

6. Data quality

Trained study coordinators and/or research associates collected clinical data from the patient's medical record onto a paper case report form. A separate research associate entered these data into a secure, web-based data management system hosted by the University of Louisville Division of Infectious Diseases. Data quality issues identified by the research associate entering the data were fixed prior to submission of the case to the database. The database also contains several data quality checks to limit out-of-range errors and inappropriate data types. These data quality structures were built in based on our decades of experience collecting and entering clinical data into electronic databases. After all data queries were resolved, the case was accepted into the database for analysis.

7. Additional Supplemental Tables and Figures

Table S2. Patient characteristics of all patients, split by study population status

	Patients consented and in whom urinary antigen performed	Patients without consent or urinary antigen performed	P-value
n	5402	2882	<0.001
Age (median [IQR])	67 [56, 78]	70.00 [57.00, 82.00]	0.001
Male sex (%)	2568 (47.5)	1257 (43.6)	0.231
Black or African American race (%)	1056 (19.5)	596 (20.7)	<0.001
Obese (%)	2030 (37.6)	844 (29.3)	0.923
HIV (%)	87 (1.6)	48 (1.7)	0.275
Neoplastic disease (%)	751 (13.9)	375 (13.0)	<0.001
Renal disease (%)	1510 (28.0)	952 (33.0)	<0.001
Congestive heart failure (%)	1640 (30.4)	855 (29.7)	0.529
Chronic obstructive pulmonary disease (%)	2765 (51.2)	1249 (43.3)	<0.001
Stroke (%)	656 (12.1)	434 (15.1)	<0.001
Current smoker (%)	1814 (33.6)	779 (27.0)	<0.001
Diabetes (%)	1758 (32.5)	969 (33.6)	0.332
Direct ICU admission (%)	913 (16.9)	545 (18.9)	0.024
Altered Mental Status (%)	812 (15.0)	762 (26.4)	<0.001
Vasopressors on first day of admission (%)	130 (2.4)	109 (3.8)	<0.001
Mechanical ventilator on first day of admission (%)	739 (13.7)	419 (14.5)	0.298
PSI Risk Class IV-V (%)	3144 (58.2)	1930 (67.0)	<0.001

Table S3. Patient characteristics of patients consented and in whom Urinary antigen detection (UAD) was performed, split by pneumococcal pneumonia

	Patients with *S. pneumoniae* identified	Patients without *S. pneumoniae* identified	P-value
n	708	4694	
Age (median [IQR])	65 [56, 75]	67 [56, 79]	0.007
Male sex (%)	323 (45.6)	2245 (47.8)	0.291
Black or African American race (%)	123 (17.4)	933 (19.9)	0.130
Obese (%)	238 (33.6)	1792 (38.2)	0.021
HIV (%)	21 (3.0)	66 (1.4)	0.004
Neoplastic disease (%)	86 (12.1)	665 (14.2)	0.165
Renal disease (%)	184 (26.0)	1326 (28.2)	0.228
Congestive heart failure (%)	199 (28.1)	1441 (30.7)	0.176
Chronic obstructive pulmonary disease (%)	403 (56.9)	2362 (50.3)	0.001
Stroke (%)	85 (12.0)	571 (12.2)	0.953
Current smoker (%)	317 (44.8)	1497 (31.9)	<0.001
Diabetes (%)	214 (30.2)	1544 (32.9)	0.171
Direct ICU admission (%)	144 (20.3)	769 (16.4)	0.010
Altered Mental Status (%)	96 (13.6)	716 (15.3)	0.263
Vasopressors on first day of admission (%)	25 (3.5)	105 (2.2)	0.050
Mechanical ventilator on first day of admission (%)	100 (14.1)	639 (13.6)	0.756
PSI Risk Class IV-V (%)	407 (57.5)	2737 (58.3)	0.709

Figure S2. Identification of *S. pneumoniae* by sample type for the study population (n=5,402)

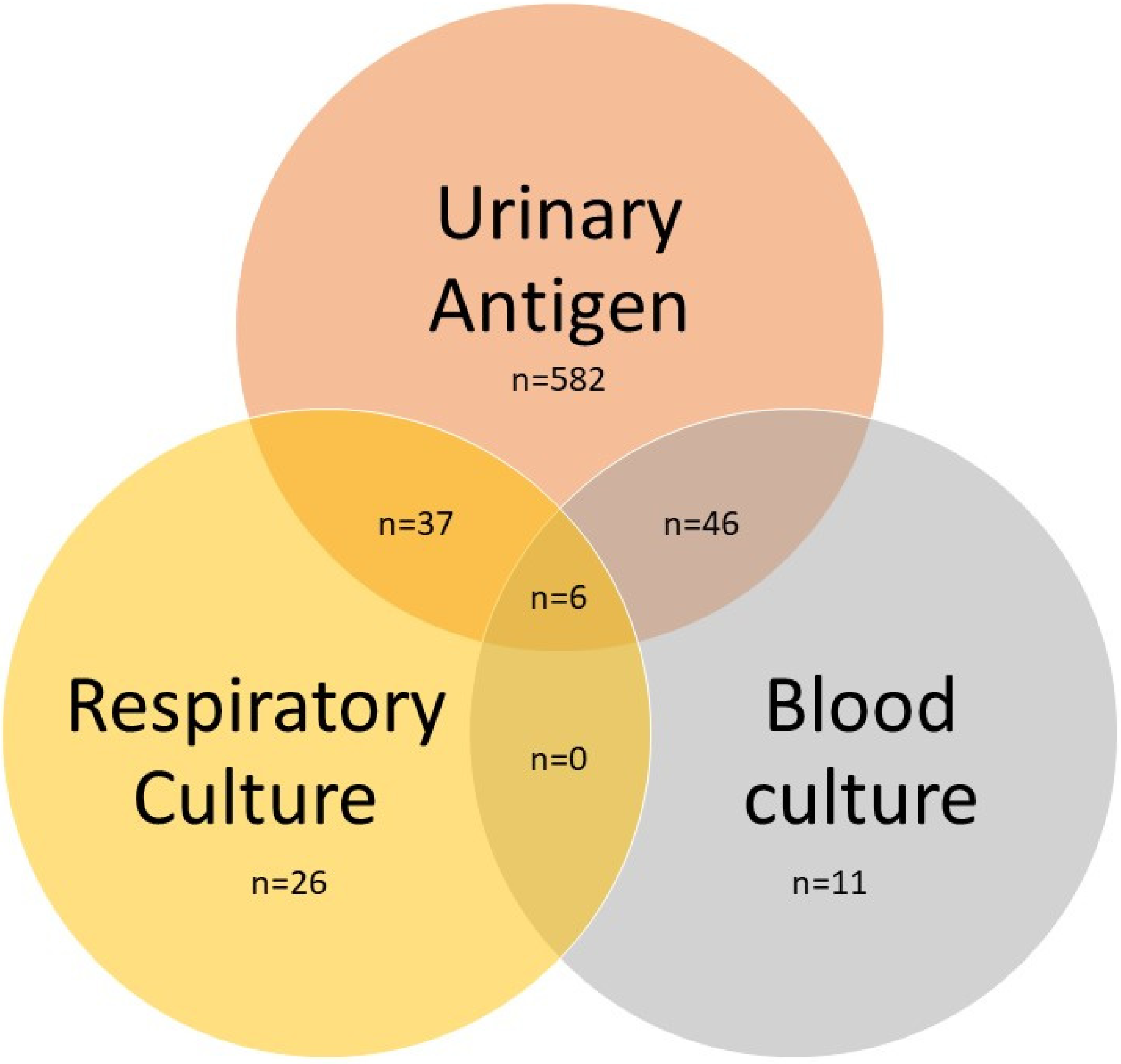

Supplement: Supplementary file 1 [file microorganisms-11-02813-s001.zip › microorganisms-2701826-supplementary.pdf]
